# Association of femoral retroversion and out-toeing gait with development of hip osteoarthritis and treatment: a systematic review

**DOI:** 10.2340/17453674.2025.43475

**Published:** 2025-04-17

**Authors:** Christos TSAGKARIS, Thomas DREHER, Patrick ZINGG, Matthias RÜGER

**Affiliations:** 1 Department of Pediatric Orthopedics and Traumatology, University Children’s Hospital, University of Zurich, Zürich; 2 Pediatric Orthopedics, Balgrist University Hospital, University of Zurich, Zürich; 3 Department of Orthopedics, Balgrist University Hospital, University of Zurich, Zürich; 4 Laboratory for Bone Biomechanics, Institute for Biomechanics, ETH Zürich, Zürich, Switzerland

## Abstract

**Background and purpose:**

Femoral retroversion (FR) is known to be a predisposing factor for femoro-acetabular impingement and is hypothesized to constitute a risk factor for early osteoarthritis of the hip. We performed a systematic review to comprehensively evaluate the evidence for FR being associated with hip osteoarthritis (OA) and the results of early treatment among adolescents and young adults.

**Methods:**

A systematic literature search was conducted in biomedical databases (PubMed, Scopus, Web of Science, Cochrane, Google Scholar) from 1970 to 2023. Inclusion criteria were studies in English or German. Studies were selected based on predefined criteria and according to PRISMA guidelines.

**Results:**

Of 37 initial records, 11 studies were included, involving 1,807 patients and 785 cadavers. Most studies were conducted in North America (7), Europe (3), and Australia (1). Diagnostic modalities included clinical examination, radiography, computed tomography, and magnetic resonance imaging. Findings from preclinical and clinical studies suggest an association between FR and hip pain, impingement, and OA. Studies reported that 5–11% of patients requiring total hip replacement exhibited FR and emphasized pain in young adults as a prompt for torsional assessment. However, contradictory results regarding the need for surgical correction were found. Surgery appears effective in relieving hip pain. Concerns exist regarding spontaneous correction at a young age and the risk of overcorrection with surgery.

**Conclusion:**

Our review underscores the lack of evidence regarding FR as a risk factor for hip osteoarthritis, and contradictory results regarding the need for surgical correction were found.

Femoral retroversion (FR), also known as coxa retrotorta, is a torsional deformity, characterized by an increased retroversion of the femoral neck relative to the femur condyles. This may be accompanied by an out-toeing gait [1,2]. Regarding its etiology, FR may be developmental resulting from or manifesting in the form of external rotation contracture of the hip during intrauterine development [3,4]. It may also be acquired as a posttraumatic torsional deformity [5]. It has been included in the 10th Revision of the International Classification of Diseases (ICD-10) under the code Q65.9 [6].

The epidemiology of FR is unclear. A study that investigated femoral version in a general population treated for femoral trauma found that FR was prevalent among white males residing in the United States of America (21.4%), but did not report the overall prevalence in other demographics [7]. Conversely, only 5% of patients referred to a tertiary center for hip pain were found to have FR [8]. According to a study focusing on patients with femoroacetabular impingement, the frequency of FR (femoral version [FV] < 10°) in patients with cam-type femoroacetabular impingement was up to 42% [9]. A study investigating patients with slipped capital femoral epiphysis indicated that FR was present in 91% to 100% of the study participants depending on the measurement method used [10], although another study in the same population indicated that FR is present in approximately 54% of patients with slipped capital femoral epiphysis [8,11].

Late-onset walking in early childhood, persistent out-toeing gait, functional limitations in sports, and knee pain comprise the most common FR manifestations. Physical examination involves assessing gait patterns, frontal plane deformities, measuring femoral version, evaluating hip range of motion, and measuring the femoral and tibial torsion. The development of painful hip pathologies on the grounds of FR can be further assessed through the FABER test and the anterior impingement test [12,13]. Computed tomography (CT) is considered the gold standard for the diagnosis of FR [14]. Magnetic resonance imaging (MRI) has also been shown to be reliable for this [11]. Three-dimensional gait analysis provides a detailed account of compensatory movements and muscle imbalances deriving from FR [15].

Treatment strategies for FR vary according to age and symptom severity. Surgical correction is rarely performed before puberty [16]. Surgical correction options encompass derotational femoral osteotomy, percutaneous femoral derotation osteotomy, and acetabular reconstruction, predominantly if an acetabular deformity is also present [17-19]. Corrections of this type have been reported in adults as well as children and adolescents [8,11,20,21].

Diagnosis and treatment of FR are for the moment subject to individual judgment in the lack of good evidence and uniform guidelines [22]. While important background understanding of the etiology and epidemiology of the condition is missing, relevant clinical questions such as the risk of developing hip osteoarthritis at an early age, a standardized method to evaluate this risk, and the need to perform corrective surgery remain unclear [10,23,24].

We evaluated the evidence for FR being (i) a risk factor associated with hip osteoarthritis, (ii) an indication for surgery and to summarize early treatment outcomes.

## Methods

A systematic literature review was conducted. Biomedical databases (PubMed, Scopus, Web of Science, Cochrane, and Google Scholar) were searched. The following search prompt was used: “((coxa retrotorta OR retroverted hip OR femoral retroversion OR “reduced femoral torsion”) AND (treatment options OR management OR therapeutic approaches OR surgical interventions OR osteotomy OR osteoarthritis) AND pediatric).” The search was conducted and reported according to the Preferred Reporting Items for Systematic Reviews and Meta-Analyses (PRISMA) Extension for systematic reviews [25].

Clinical and pre-clinical research, case reports, and conference abstracts published in English or German from databases’ inception to the May 30, 2023 were considered for inclusion. Exclusion criteria covered secondary research, inaccessible full-text, retracted studies, and pre-prints. Conference papers were considered based on the assumption that they were accepted for presentation through peer review.

Results of searches were imported into Rayyan AI (https://www.rayyan.ai/), an AI-powered tool for systematic literature reviews, and duplicates were eliminated. The reviewer conducted a preliminary screen to exclude any off-topic studies and articles followed by a detailed screening of all titles and abstracts. The full texts of potentially eligible studies were retrieved. After the final selection of studies, an additional manual search was performed in the literature lists of the included studies.

The bibliographic metadata (RIS) of the studies, including titles, author details, abstract, and keywords, that were deemed appropriate for full-text assessment and the studies that were eventually included were also uploaded to VOSviewer (version 1.6.15, University of Leiden) and subjected to automated network term analysis.

### Ethics, data sharing plan, funding, use of AI, and disclosures

The present paper consists of a literature review based on publicly available information published in peer-reviewed journals; all reported studies have been cited. No primary data was collected or generated and no human or animal subjects could be identified. Therefore, Institutional Review Board (IRB) clearance was not deemed necessary. Software incorporating AI features has been used for storing and selecting literature (Rayyan AI), managing and formatting references (Mendeley, 2.98.0© 2023; Mendeley Ltd, Elsevier, London), and to grammatically polish parts of the text (ChatGPT, OpenAI, GPT-4). The authors have manually checked the results of these processes. No funding was acquired and the text has been manually prepared. The authors have no relevant conflict of interest to declare to the best of their knowledge. Complete disclosure of interest forms according to ICMJE are available on the article page, doi: 10.2340/17453674.2025.43475

## Results

### Quantitative overview

The literature search yielded 37 records from electronic databases. After removing duplicates and screening abstracts and titles, 13 records remained (Figure). Following a full-text assessment based on inclusion and exclusion criteria and an additional search for eligible studies in the reference lists of included records, 11 studies were selected (Table 1). Included preclinical (cadaveric or in silico studies, n = 3) and clinical studies reported on 1,807 patients and 785 cadavers. The majority of the studies were conducted in North America (n = 7), followed by Europe (n = 3) and Australia (n = 1) .

**Table 1:** Detailed presentation of the studies included

Authors, date	Study type	Summary	Population	Mean age	% female	Modality	Follow-up	Outcome (FR is ...)
Vallon, 2015	Preclinical	Reduced femoral torsion, < 10° was associated with impingement at the anterosuperior rim area	3D model of the hip joint	NA	NA	Finite elements modeling	NA	
Bourget-Murray, 2021	Preclinical	FR can increase the risk for FAI in patients undergoing hip resurfacing arthroplasty (HRA)	60 simulations	NA	NA	Finite elements modeling	NA	A pre-arthritic condition
Wang, 2022	Preclinical	Frequency of FR among a cadaveric collection	579 cadavers	55.9	86	CT, computational, biomech. testing	NA	A pre-arthritic condition
Meier, 2023	Clinical	Femoral version variations among patients with DDH and AR	333 patients (384 hips)	29	27	CT, MRI	NA	A pre-arthritic condition
Pierrepont, 2019	Clinical	Femoral version irregularities among adults who had undergone THR	1,215 patients	63	51	CT	NA	A pre-arthritic condition
Tönnis, 1991	Clinical	Outcomes of rotational osteotomies in a case series of children and adults with diminished femoral antetorsion	59 patients (111 hips)	21	NA	Radiography, CT	NA	A pre-arthriotic condition An indication for surgery
Thawrani, 2017	Clinical	The coexistence of FR with acetabular retroversion comprises an indication for reverse PAO, while traditional PAO is indicated in children and young adults with isolated FR	10 patients (12 hips)	33.8	NA	Radiography	NA	An indication for surgery
Lall, 2019	Clinical	Patients with FR and FAI benefit from HA resolving FAI	59 patients	37.4	67	MRI	5 y	An indication for surgery
Kelly, 2012	Clinical	Arthroscopic cam decompression in young adults with FAI contributes to normalizing version anomalies among patients with FA and FR	55 patients (56 hips)	27.4	80	Preoperative CT, Postoperative radiography	3 m	An indication for surgery
Morris, 2019	Cadaveric	Increased femoral anteversion can lead to FAI faster than reduced femoral anteversion or FR	206 cadavers	NA	91.3	Computational, biomechanical testing	NA	No indication for surgery
Ringling, 2021	Clinical	Arthroscopic femoral rotation osteotomy in symptomatic individuals leads to subjective relief from pain and motion impairment without causing objective patellofemoral instability or modifying the patello-femoral geometry	23 patients, (25 hips, 18 with decreased FT (≤4°))	24	40	Clinical (objective and subjective) assessment, MRI	37 m	An indication for surgery
Pitkow, 1975	Clinical	FR is a rare cause of out-toeing gait and when present tends to resolve automatically at a young age	Pediatric population	NA	NA	Clinical assessment, radiography	NA	No indication for surgery
Dora, 2002	Clinical	In young adults with combined femoral and acetabular retroversion, undergoing pelvic osteotomy in combination with derotational femoral osteotomy, the postoperative remodeling of femoral neck torsion during growth may not compensate for the retroversion of the acetabular dome	73 patients (97 procedures)	4.8	11	Radiography	11.5 y	No indication for surgery

NA: Not applicable or not available.

**Figure:**
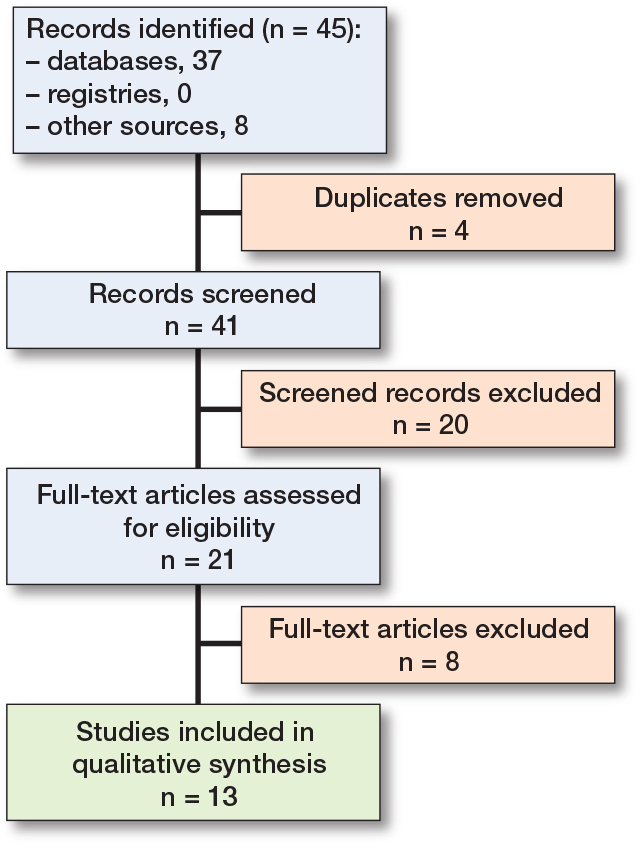
Flow diagram of the systematic literature search based on predefined criteria and according to PRISMA guidelines.

The studies address 2 main themes; (a) whether FR is a condition predisposing to early development of hip osteoarthritis and (b) whether surgical correction is indicated or contraindicated. The bibliometric analysis indicated the terms impingement, correction, and pelvic osteotomy as most prevalent among the initially selected studies, and the terms retroversion, acetabular dome, and hip as the most prevalent among the eventually included studies. Among the 8 clinical studies included, 2 were prospective—1 a completed clinical trial and 1 a midterm analysis [26,27]—while the rest were retrospective. Due to methodological heterogeneity and the lack of a standardized appraisal tool for non-randomized studies, a reliable quality assessment was not feasible [28].

### Definition and diagnosis of femoral retroversion and coexistent hip pathologies

Radiography and CT scans were the most common diagnostic modalities for FR (n = 10), followed by MRI (n = 2) and clinical examination (n = 1). A number of preclinical (n = 2) and clinical studies (n = 4) utilized a combination of diagnostic methods [26,29-33]. Clinical studies used slightly different definitions of FR. A number of them abided by Tönnis and Heinecke [31] defining FR as femoral version lower than 10° at the time of the completion of skeletal development [27,34]. Thawrani and colleagues [37] set 15° as a cutoff, while Kelly et al. [26] and Pierrepont et al. [35] set the limit at 5° and 0° for the same time point, respectively. Pitkow, in his inaugural study conceptualizing FR [32], followed a different approach, setting a different approximate physiological femoral version for newborns and children aged 1, 5, and 15 years (Table 2).

**Table 2:** Diagnostic methods and cutoffs for femoral retroversion, additional version measurements, and respective surgical techniques

Study	Modality	Cutoff value	Additional version assessment	Surgical correction	Remarks
Meier, 2023	CT, MRI	< 10°	Acetabulum	PAO + DFO or HA	HA in the case of FAI
Pierrepont, 2019	CT	< 0°	None	Total hip replacement	Non-reversible pathology
Tönnis, 1991	Radiography, CT	< 10°	None	DFO	
Thawrani, 2017	Radiography	< 15°	Acetabulum	Reverse PAO	Reverse PAO in case of coexisting acetabular and femoral retroversion
Lall, 2019	MRI	< 10°	Acetabulum	HA	HA in the case of FAI or LT
Kelly, 2012	Preoperative CT, postoperative radiography	< 5°	Acetabulum	HA	HA in the case of FAI and LT
Pitkow, 1975	Clinical, radiography	< 35° at birth, < 30° at 1 year, < 25° at 5 years, < 15° at 15 years	None	None	
Dora, 2002	Radiography	< 10°	Acetabulum	Salter or triple pelvic osteotomy combined with DFO	

CT: computed tomography, MRI: magnetic resonance imaging, PAO: periacetabular osteotomy, DFO: derotational femur osteotomy, HA: hip arthroscopy, FAI: femoroacetabular impingement, LT: labral tears.

### Femoral retroversion as risk factor for the early development of hip osteoarthritis

The results related to FR as a risk factor for the development of hip osteoarthritis at an early age include findings from both preclinical and clinical studies. Computational modeling revealed that progressive FR leads to increased risk of impingement, which may occur earlier in the flexion range of motion (ROM) [21,36]. Additionally, it was observed that the distal femur trochlear groove may have limited capacity to adjust for femoral deformities caused by FR, resulting in changes in the configuration of the lower extremity and an increased risk of hip degeneration [30].

In clinical studies, it was found that up to 12% of developmental dysplasia of the hip (DDH) patients, particularly females, exhibited FR in combination with acetabular retroversion, which increases the risk of anterior hip pain and degeneration [34]. Furthermore, FR was associated with pain in individuals beginning before the age of 30, emphasizing the significance of early detection [31]. The significance of early detection and operation was further highlighted by a study reporting an FR incidence of 11% in males and 5% in females requiring total hip replacement (vs 14% incidence of femoral anteversion in the same demographic) [35].

### Need for surgical correction of femoral retroversion

Studies that favored surgical correction on the grounds of FR demonstrated that rotation femoral osteotomy could lead to pain relief and improved gait in patients with pain-evoking FR [31]. This was particularly relevant for patients with coexisting femoral and acetabular retroversion, which can be corrected with better functional and clinical outcomes by means of reverse periacetabular osteotomy (PAO) rather than conventional PAO according to Thawrani and colleagues [37]. Surgical corrections are further supported by studies which showed that favorable hip rotation outcomes can be comparable across patients with decreased, normal, or increased femoral version [20,27]. It appears that patients with FR can also benefit from operations correcting cam abnormalities. Kelly and colleagues (2012) reported that patients with FR who underwent arthroscopic cam decompression on the grounds of symptomatic hip impingement exhibited normalization of radiographic (alpha angle) and clinical (hip rotation) outcomes, although derotation was not the primary goal of this operation [26]. Therefore, surgery addressing either FR or comorbid conditions such as acetabular retroversion of hip impingement seems to result in favorable functional and radiographic outcomes.

Concerns regarding the surgical correction of FR were raised by studies highlighting the tendency for spontaneous correction potential at a young age or the risk of corrective surgery leading to suboptimal results. In one of the first clinical studies on FR, Pitkow (1975) concluded that FR tends to resolve by 18 months of age in the majority of patients, rendering very early surgical correction unnecessary and supporting the idea that correction may still be achieved—at a lower rate—at an older age [32]. On top of this, Dora and colleagues (2002) indicated that in patients with combined femoral and acetabular retroversion, undergoing pelvic osteotomy (Salter or triple pelvic osteotomy) in combination with derotational femoral osteotomy, the postoperative remodeling of femoral neck torsion during growth may not compensate for—and may even aggravate—the retroversion of the acetabular dome [38].

### Surgical techniques for the correction of femoral retroversion and coexistent pathologies

The surgical techniques employed in the aforementioned studies and their indications are presented in Table 2. In principle, surgical decision-making depends on the presence of a hip pathology and/or an acetabular deformity. Femoroacetabular impingement (FAI) is treated by means of hip arthroscopy (HA) rather than derotational femur osteotomy (DFO), if the etiology of most or all of the FAI is intraarticular [26,27,33]. In the latest studies, DFO is combined with either PAO or reverse PAO in the case of coexistent acetabular retroversion, with the latter being recommended as optimal treatment for this demographic in contrast to conventional PAO, which is prioritized for patients with isolated acetabular retroversion [33,37]. In the past, it appears that DFO would be combined with either Salter or triple osteotomy rather than PAO.

## Discussion

We showed that there is a lack of evidence concerning FR as a risk factor for hip OA and contradictory results regarding the need for surgical correction.

The pre-arthritic potential of FR has been supported by preclinical studies, involving either computational simulation or cadaveric collections (n > 500) [30,37]. Clinical studies report FR in patients presenting or operated on for hip osteoarthritis or pre-osteoarthritic conditions, such as FAI [31,33,35]. However, research reporting follow-up of patients with FR from a young age until the development of hip pathologies is limited. Patients participating in clinical studies do not receive a standardized evaluation of concomitant version irregularities affecting the acetabulum or the tibia and therefore understanding biomechanical risk is challenging. Moreover, gait analysis is rarely used for the diagnosis of occult FR. Consequently, an etiological relation between FR and hip pathologies can be assumed, but remains in question.

Hip preservation or replacement surgery is performed on patients with FR on the grounds of established hip pathologies and functional limitations [26,27,31,37]. FR is, however, often not a primary research outcome of studies reporting these operations. Patients received surgery to address coexisting conditions of FR or, in the best-case scenario, progression of FR to hip pain. The only relevant clinical trial reported potentially beneficial effects of hip impingement corrective surgery on hip mobility limitations associated with an underlying FR [26]. This indicates that surgery does not harm when the damage is already established, but provides few, if any, insights to the potential for early correction of FR to prevent progression of FR to painful hip conditions. Surgical indications in asymptomatic or oligosymptomatic patients could not be identified.

Studies that opposed surgery presented a twofold argumentation. On one hand they emphasized the correction potential of hip deformities in children, but they did not provide evidence that this is clinically relevant in children older than 18 months [32]. On the other hand, they documented suboptimal clinical results or predicted them assuming a high risk of iatrogenic overcorrection [29,38]. While the concerns of Dora et al. (2002) are concrete, it appears that bad outcomes were common among patients with coexisting acetabular version deformities [38]. This remains a valid concern, given that femoral and acetabular retroversion tend to coexist in approximately 12% of patients with hip impingement [9,33]. The study by Thawrani et al. seconds this concern by recommending detailed preoperative evaluation for both femoral and acetabular version and recommends reverse PAO to improve the relevant surgical outcomes [37]. Moreover, overcorrection seems to persist in approximately half of the patients undergoing femoral osteotomies with deliberate or accidental overcorrection [28,39]. This underscores the lack of clear surgical indications in the pediatric population, where development might interfere with postoperative remodeling, and calls for long-term follow-up of children already operated on for FR. Analyzing the gait development postoperatively is necessary to further comprehend the relevance of the concerns raised by Morris et al. and Dora et al. [29,38].

The discrepancies among diagnostic cutoffs of FR and imaging modalities guiding surgical—or non-surgical—decision-making also need to be considered. Despite the wide acceptance of the definition of Tönnis et al., some studies adopt higher and lower cutoffs, creating variations in the number of patients eligible for evaluation and potentially surgery (see Table 2). Despite this, a growing number of studies have stressed the consistency between CT and MRI measurements of femoral version [34,40,41]. Therefore, although different definitions have been followed, the absolute numbers used can be considered to a great extent consistent despite the different imaging modalities employed. A commonly accepted cut-off of FR, potentially < 10°, constitutes a prerequisite for comparable evidence.

Additional concerns surround the geographical distribution of studies and their respective population coverage. The geographic distribution of the clinical studies in Europe and North America indicates that the presented evidence is aligned with the existing epidemiological observations on white Caucasians and rarely includes members of different demographic groups.

### Limitations

Different types of operations, namely reverse and traditional PAO, arthroscopic cam decompression, rotational femur osteotomies, and total hip arthroplasties, were examined and were performed under a wide range of indications from trauma to FAI and hip osteoarthritis. Moreover, it is likely that studies which used different terminologies or definitions not identified during the search—the terminologies around FR have certainly changed since the description of “external rotation contracture of the extended hip” by Pitkow in 1975 or “diminished femoral anteversion” by Tönnis and Heinecke in 1991 [31,32]. On these grounds, an etiological connection between FR and the development of either FAI or hip osteoarthritis could not be asserted. Regarding evidence appraisal, a tool developed particularly for pediatric surgery could not be used due to the inclusion of studies with adult patients [42,43]. Finally, it needs to be disclosed that a protocol for the study has not been registered with PROSPERO and has therefore not been accessible to other researchers.

### Conclusions

We showed that there is a lack of evidence concerning FR as a risk factor for hip OA and contradictory results regarding the need for surgical correction. In perspective, follow-up of the hip’s rotational profile, timely imaging and identification of coexisting deformities may be crucial for improving long-term outcomes.

## Notes

CT: literature search and selection, writing, formatting, revision; TD: conceptualisation, critical revision, supervision; PZ: critical revision, supervision; MR: literature search, critical revision, supervision.

Handling co-editors: Ivan Hvid and Robin Christensen

*Acta* thanks Per Kjærsgaard-Andersen and Michael Brian Millis for help with peer review of this manuscript.
